# MODELING REACTIVATED HETEROCHROMATIN USING AN AGING 3D CELL MODEL

**DOI:** 10.1093/geroni/igac059.103

**Published:** 2022-12-20

**Authors:** Simone Sidoli, Stephanie Stransky, Ronald Cutler, Sarah Graff, Jennifer Aguilan

**Affiliations:** Albert Einstein College of Medicine, New York, New York, United States; Albert Einstein College of Medicine, New York, New York, United States; Albert Einstein College of Medicine, New York, New York, United States; Albert Einstein College of Medicine, New York, New York, United States; Albert Einstein College of Medicine, New York, New York, United States

## Abstract

Chromatin state and dynamics is modulated by chromatin interacting proteins, in particular histones and their post-translational modifications. We investigate the chromatin-bound proteome in aging to understand how DNA readout is misregulated in senescent cells. We focus on domains of reactivated heterochromatin, i.e. chromatin domains undergoing anomalous decondensation and decorated by histones co-modified with markers of condensed heterochromatin and active transcription. We optimize our mass spectrometry methods 3D cell models, and then apply them on B-cells retrieved from Ashkenazi Jews donors. In this cohort, we particularly focus on offsprings of exceptional longevity, i.e. individuals in their 70s with projected longer lifespan due to their centenarians ancestors. Preliminary data on 60 individuals revealed the enrichment of unusual histone modifications in the OPEL group, the group we utilize as indicator of projected longer lifespan. We have then applied biochemistry techniques to identify the role of these unusual modifications on chromatin readout.

